# YCu(TeO_3_)_2_(NO_3_)(H_2_O)_3_: a novel layered tellurite

**DOI:** 10.1107/S2056989016011464

**Published:** 2016-07-19

**Authors:** Stuart J. Mills, Maja A. Dunstan, Andrew G. Christy

**Affiliations:** a Geosciences, Museum Victoria, GPO Box 666, Melbourne 3001, Victoria, Australia; b School of Chemistry, University of Melbourne, Parkville 3010, Victoria, Australia; c Ocean and Climate Geoscience, Research School of Earth Sciences, Mills Rd, Australian National University, Canberra, ACT 2601, Australia

**Keywords:** crystal structure, layered arrangement, tellurite, oxysalt, stereoactive lone pair, synchrotron radiation

## Abstract

A new hydrated yttrium copper tellurite nitrate, yttrium(III) copper(II) bis­[trioxidotellurate(IV)] nitrate trihydrate, has been synthesized hydro­thermally in a Teflon-lined autoclave and structurally determined using synchrotron radiation. The new phase is the first example containing yttrium, copper and tellurium in one structure. Its crystal structure is unique, with relatively strongly bound layers extending parallel to (020), defined by YO_8_, CuO_4_ and TeO_3_ polyhedra, while the NO_3_^−^ anions and one third of the water mol­ecules lie between those layers. The structural unit consists of [Cu_2_(TeO_3_)_4_]^4−^ loop-branched chains of {Cu⋯Te⋯Cu⋯Te} squares running parallel to [001], which are linked further into layers only through Y(O,H_2_O)_8_ polyhedra. Weak ‘secondary’ Te bonds and O—H⋯O hydrogen-bonding inter­actions, involving water mol­ecules and layer O atoms, link the layers and inter­layer species. IR spectroscopic data are also presented.

## Abstract 2

A new layered tellurite has been synthesized, where the structural unit consists of [Cu_2_(TeO_3_)_4_]^4−^ loop-branched chains of {Cu⋯Te⋯Cu⋯Te} squares, which are linked further into layers only through Y(O,H_2_O)_8_ polyhedra.

## Chemical context   

Recent discoveries of a wide range of novel tellurium minerals have prompted numerous structural studies of tellurium oxysalts (Kampf *et al.*, 2013 ▸; Christy *et al.*, 2016*a* ▸). As well as the characterization of these naturally occurring minerals, various syntheses have also been undertaken as part of this ongoing study, yielding an array of new structures, including that of novel Na_11_H[Te(OH)_3_]_8_[SO_4_]_10_(H_2_O)_13_ (Mills *et al.*, 2016 ▸). Several tellurium oxide species with various yttrium oxide polyhedra present in the structure have been synthesized in the past, including compounds with both Te^IV^ and Te^VI^ atoms. Tellurium is stable in numerous oxidation states and shows large diversity in bonding (Christy & Mills, 2013 ▸). Its +IV and +VI oxidation states are of greatest inter­est in relation to naturally occurring weathering products of minerals, and are able to form a wide variety of oxide polyhedra, with TeO_3_^2−^ most prevalent (Song *et al.*, 2014 ▸). The TeO_3_^2−^ anion shows a wide variety of connectivities, with three oxido ligands and the 5*s* ^2^ electron lone pair occupying the vertices of the distorted polyhedra, and are found in a variety of layer and chain structures in inorganic compounds (Johansson & Lindqvist, 1978 ▸). This is demonstrated in compounds such as NaYTe_4_O_10_ with YO_8_ and TeO_4_ polyhedra, KY(TeO_3_)_2_ and RbY(TeO_3_)_2_ with YO_6_ octa­hedra and trigonal–pyramidal TeO_3_^2−^ anions, CsYTe_3_O_8_ with YO_6_ and TeO_4_ polyhedra (Kim *et al.*, 2014 ▸), as well as yttrium tellurium oxides with Te^VI^ atoms (Kasper, 1969 ▸; Höss & Schleid, 2007 ▸; Noguera *et al.*, 2012 ▸). As a consequence of this range of chemistry, tellurium is the most anomalously diverse element found in minerals compared to its scarcity in the earth’s crust (Christy, 2015 ▸). Many copper-containing tellurium oxides have been successfully synthesized (Feger *et al.*, 1999 ▸; Koteswararao *et al.*, 2013 ▸; Sedello & Müller-Buschbaum, 1996 ▸), and copper is also present in many tellurium-containing minerals; indeed, out of the unusually large inventory of tellurium secondary minerals at Otto Mountain, the majority contains copper (Christy *et al.*, 2016*a* ▸). Despite this, there are very few synthetic rare earth copper tellurium oxides known, and to the best of our knowledge a compound containing all three of copper, yttrium and tellurium has not been characterized so far. Although layered structures with inter­stitial ions are common for Te^IV^ compounds, nitrate is found as an anion in very few, which motivates the use of metal nitrates in the synthesis of novel tellurium oxides. The only other compounds with simple tellurite and nitrate anions whose structures have been reported to date are the layered compounds Ca_6_(TeO_3_)_5_(NO_3_)_2_ and Ca_5_(TeO_3_)_4_(NO_3_)_2_(H_2_O)_2_ (Stöger & Weil, 2013 ▸). Nitrates of polymerized Te(IV) complexes are also known. The compound AgTeO_2_(NO_3_) (Olsson *et al.*, 1988 ▸) contains an electrically neutral [Te_2_O_4_]^0^ chain (Christy *et al.*, 2016*b* ▸), while [Te_2_O_3_OH](NO_3_) contains a cationic [Te_2_O_3_OH]^+^ layer (Anderson *et al.*, 1980 ▸; Christy *et al.*, 2016*b* ▸).

## Structural commentary   

Bond-valence sums are given in Table 1 ▸. In general, the bond-valence data of Table 1 ▸ were calculated using the bond-valence parameters of Brown & Altermatt (1985 ▸), except that the Te—O data were from Mills & Christy (2013 ▸). However, Brown (2009 ▸) noted that no single pair of *r*_0_ and *b* values is adequate for O—H bonds, since O⋯O repulsion increases the length of weak O—H bonds relative to strong ones. Here, the parameterization of Yu *et al.* (2006 ▸) was used, with *r*_0_ = 0.79 Å for bond valence < 0.5 valence units, *r*_0_ = 1.409 Å for bond valence > 0.5 v.u., and *b* = 0.37 Å in both cases.

The structure of the title compound is strongly layered. Layers parallel to (020) are defined by YO_8_, CuO_4_ and TeO_3_ polyhedra, while NO_3_^−^ anions and one third of the water mol­ecules (O*W*1) lie between those layers. Tellurite and nitrate anions (involving atoms O1–O9) are clearly distinguished from water mol­ecules O*W*1–O*W*3 by their bond-valence sums (Table 1 ▸). Within the layers, Y is eightfold coordinated in a distorted snub disphenoidal (triangular dodeca­hedral) arrangement by 6 × O^2−^ and 2 × H_2_O at 2.290 (3)–2.497 (3) Å. Cu is in square-planar coordination, with four close oxygen neighbours at 1.904 (3)–1.999 (3) Å. Two more oxygen ligands at 2.811 (4) and 2.817 (4) Å complete an octa­hedron that is very elongated due to the Jahn-Teller distortion. Te1 is trigonal–pyramidally coordin­ated by three oxygen atoms at 1.883 (3)–1.911 (3) Å. Three ‘secondary bonds’ to O atoms at 2.657 (3)–2.837 (3) Å complete a polyhedron that can be described as an octa­hedron that is very distorted due to the lone-pair stereoactivity. Te2 has very similar coordination, with three primary Te—O bonds of 1.893 (3)–1.905 (3) Å and three secondary bonds of 2.681 (4)–2.798 (3) Å. In each case, two of the secondary bonds provide additional bracing within the {Y⋯Cu⋯Te} layer, while the third is to a nitrate oxygen (Te1—O7 and Te2—O8, both ≃ 2.72 Å), and thus provides weak bridging between the layers and inter­layer species. The nitrate oxygen atom O9 makes a seventh very distant ligand for both Te1 [3.231 (4) Å] and Te2 [3.350 (4) Å], further than the shortest Te⋯Cu distances and with bond valences < 0.05 valence units, using the parameters of Mills & Christy (2013 ▸).

**Figure 1:**
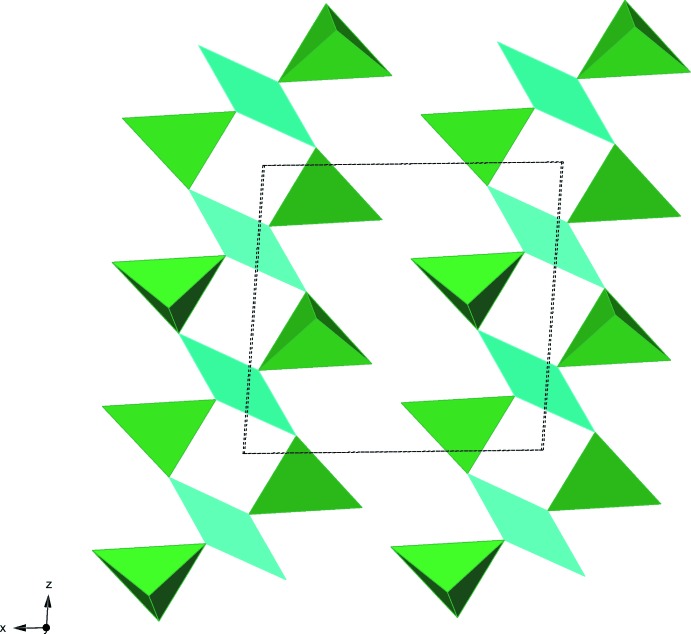
View in polyhedral mode of the [Cu_2_(TeO_3_)_4_]^4−^ loop-branched chains running parallel to [001]. CuO_4_ polyhedra are cyan, TeO_3_ polyhedra are green

**Figure 2:**
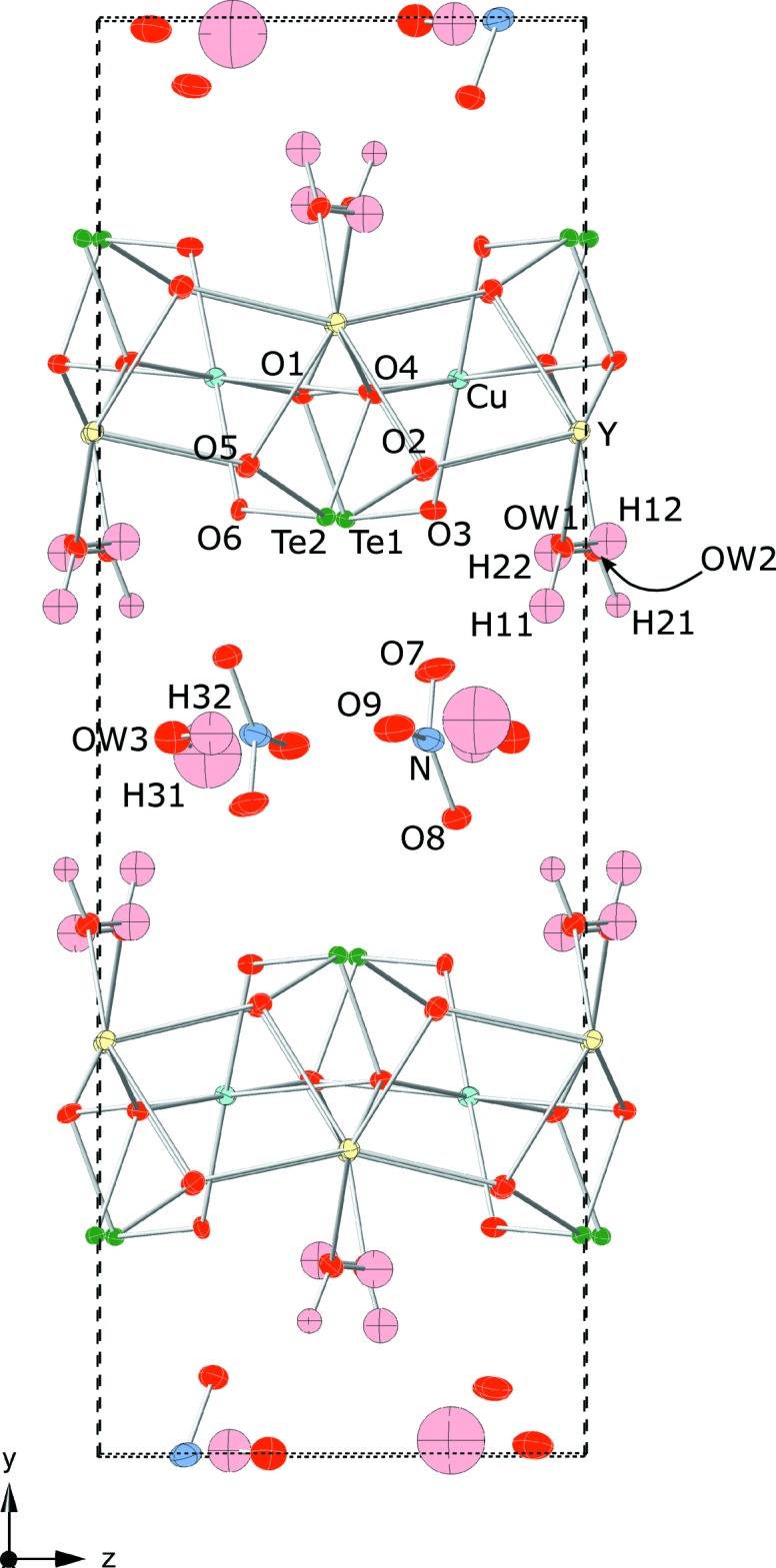
The crystal structure of YCu(TeO_3_)_2_(NO_3_)(H_2_O)_3_ viewed down [100]. O atoms are red, Y yellow, Cu cyan, Te green, N light-blue and O atoms of water mol­ecules pink. Displacement ellipsoids are drawn at the 50% probability level.

The identification and classification of a strongly bonded ‘structural unit’ (Hawthorne, 2014 ▸) in the structure of this compound depends crucially on which bonds are regarded as strong enough to define such a unit. The classification of Te oxycompound structures by Christy *et al.* (2016*b* ▸) in general used thresholds of about 2.45 Å for Te—O and 2.20 Å for Cu—O bonds, while no bonds to 8-fold coordinated cations were considered to be part of the structural unit. The same criteria applied to the current structure would regard the CuO_4_ squares as isolated from one another, although inclusion of the long Cu—O bonds would link CuO_4+2_ polyhedra to form *trans* edge-sharing chains parallel to [001]. Without the long bonds, CuO_4_ squares are linked to their neighbours most strongly *via* TeO_3_ pyramids, to produce loop-branched chains [Cu_2_(TeO_3_)_4_]^4−^ of {Cu⋯Te⋯Cu⋯Te} squares running parallel to [001] (Fig. 1 ▸). These chains are the structural units, since they are linked further into layers only through Y(O,H_2_O)_8_ polyhedra (Fig. 2 ▸). It is noteworthy that this chain is similar in topology but not in geometrical configuration to the structural unit of Dy[CuCl(TeO_3_)_2_] and its Er—Cl and Er—Br analogues (Shen & Mao, 2005 ▸). However, in the current compound, the {Cu⋯Te} squares are non-planar, so that the chain periodicity is doubled, and Cu does not have chloride as an additional ligand. Furthermore, in the structures of the compounds of Shen and Mao (2005 ▸), rare earth cations link the chains into a three-dimensional framework rather than into layers.

H11, H12, H22 and H31 were found to make relatively strong hydrogen bonds (Table 2 ▸) to respectively O*W*3, O6, O3 and O7 at distances between 1.88–1.96 Å. H12 and H31 have additional acceptor O atoms at greater distances, respectively O4 at 2.59 Å and O8 at 2.54 Å. The remaining H atoms each have two oxygen neighbours at greater distances, suggesting weak bifurcated hydrogen bonding: O*W*3 at 2.23 Å and O8 at 2.40 Å for H21, and O8 at 2.44 Å, O9 at 2.64 Å for H32.

The layers of the structure are linked by only weak bonds. The bridges Te1⋯O7—N—O8⋯Te2 mentioned above have Te⋯O ≃ 2.72 Å, implying a bond of 0.15 valence units (Mills & Christy, 2013 ▸). The hydrogen bonds in the bridges O*W*1—H11⋯O*W*3⋯H21—O*W*2 are of comparable bond valence.

It is noteworthy that the IR spectrum shows three distinct O—H bands at 3460, 3145 and 2900 cm^−1^. According to Libowitzky (1999 ▸), this would be typical for O—H⋯O distances of ∼ 2.83, 2.69 and 2.63 Å. The first two of these are broadly consistent with the O⋯O distances for the strongest hydrogen bonds indicated by the refinement: O*W*1—H12⋯O*W*3 = 2.85 Å, O*W*2—H12⋯O3 = 2.74 Å and O*W*1—H11⋯O6 = 2.73 Å. However, the band at 2900 cm^−1^ is lower in frequency than would be expected.

## Spectroscopy   

The infrared spectrum was obtained using a Bruker Alpha FTIR with a diamond Attenuated Total Reflectance attachment (ATR), DTGS (Deuterated Triglycine Sulfate) detector, 4 cm^−1^ resolution and 4000–450 cm^−1^ range. The samples were placed on the ATR crystal and pressure exerted by screwing the pressure clamp onto the sample to ensure maximum contact with the ATR crystal. 128 scans were taken for each item and co-added. Band assignments are consistent with those given in Kampf *et al.* (2013 ▸). Numerical values of the spectrum and assignments of the vibration bands are given in Table 3 ▸; the spectrum is deposited as a supplementary figure.

## Synthesis and crystallization   

Dark blue prisms of YCu(TeO_3_)_2_(NO_3_)(H_2_O)_3_ were synthesized hydro­thermally. For the synthesis, Y(NO_3_)_3_·6H_2_O (Aldrich, 99.8%), Cu(NO_3_)_2_·3H_2_O (Sigma–Aldrich ≥99%) and Te 200 mm mesh (Aldrich, 99.8%) were used as starting materials. A 1:1:1 molar ratio of the reagents in 20 ml water was reacted in a Teflon autoclave bomb at 473 K for 3 days. Crystals of YCu(TeO_3_)_2_(NO_3_)(H_2_O)_3_ were separated manually from a blue powder of undetermined composition in a few percent yield. Several unsuccessful attempts were made to synthesize YCu(TeO_3_)_2_(NO_3_)(H_2_O)_3_ from a stoichiometric mixture of the reagents, using the molar ratio 1:1:2. We also were unsuccessful in producing new compounds, with the same structure type or not, using La, Ce, Nd or Gd in place of Y.

## Refinement   

Single crystal X-ray diffraction experiments were carried out on the micro-focus macromolecular beam line MX2 of the Australian Synchrotron. Details of data collection and structure refinement are provided in Table 4 ▸. Hydrogen atoms H11, H12 and H21 were located during refinement as difference peaks of about one e^−^ / Å^3^ occurring at a distance of *ca*. 0.9–1.0 Å from their nearest oxygen atom. In all cases, short O—H bonds were directed towards another oxygen atom, indicating the existence of hydrogen bonds. Positions were estimated for the remaining hydrogen atoms, assuming water mol­ecule O—H distance near 0.9 Å, H—O—H bond angle near 104°, that O—H vectors were directed to make hydrogen bonds to nearby oxygen atoms, if possible, and that the arrangement of O—H and O⋯H around O*W*3 was approximately tetra­hedral. In all cases, residuals of > 0.6 electrons were found close to the expected positions, that could be identified with the H atoms. H positions were finally included in the refinement, assuming full occupancy, isotropic displacement parameters were fixed to 1.5× of their corresponding O atom and the O—H distance was restrained at 0.90 (3) Å.

## Supplementary Material

Crystal structure: contains datablock(s) I, global. DOI: 10.1107/S2056989016011464/wm5307sup1.cif

Structure factors: contains datablock(s) I. DOI: 10.1107/S2056989016011464/wm5307Isup2.hkl

Supporting information file. DOI: 10.1107/S2056989016011464/wm5307sup3.pdf

CCDC reference: 1493330

Additional supporting information: crystallographic information; 3D view; checkCIF report

## Figures and Tables

**Table 1:** Bond-valence sums (in valence units) for YCu(TeO_3_)_2_(NO_3_)(H_2_O)_3_

	**Y1**	**Cu1**	**Te1**	**Te2**	**N1**	**H11**	**H12**	**H21**	**H22**	**H31**	**H32**	**Σ**	**Σ**(excluding H)
**O1**	0.401	0.544, 0.047	1.128									**2.12**	**2.12**
**O2**	0.478, 0.275		1.145	0.130								**2.03**	**2.03**
**O3**		0.436	1.208	0.173					0.232			**2.05**	**1.82**
**O4**	0.399	0.534, 0.046		1.148			0.041					**2.17**	**2.13**
**O5**	0.481, 0.316	0.421	0.118	1.165								**2.08**	**2.08**
**O6**			0.183	1.179			0.279					**2.06**	**1.78**
**O7**			0.156		1.562							**1.72**	**1.72**
**O8**				0.156	1.609			0.068		0.047		**1.88**	**1.77**
**O9**					1.712						0.062, 0.036	**1.81**	**1.71**
**O*W*1**	0.384					0.755	0.755					**1.89**	**0.38**
**O*W*2**	0.389							0.771	0.769			**1.93**	**0.39**
**O*W*3**						0.224		0.110		0.743	0.761	**1.84**	**0.00**
**Σ**	**3.12**	**2.03**	**3.93**	**3.94**	**4.88**	**0.98**	**1.08**	**0.95**	**1.00**	**0.79**	**0.86**		

**Table 2:** Hydrogen-bond geometry (Å, °)

*D*—H⋯*A*	*D*—H	H⋯*A*	*D*⋯*A*	*D*—H⋯*A*
O*W*1—H11⋯O*W*3	0.89 (3)	1.96 (3)	2.854 (6)	174 (7)
O*W*1—H12⋯O6^i^	0.89 (3)	1.88 (4)	2.729 (5)	157 (7)
O*W*2—H21⋯O8^i^	0.89 (3)	2.41 (6)	3.074 (5)	132 (6)
O*W*2—H21⋯O*W*3^ii^	0.89 (3)	2.22 (5)	2.949 (6)	139 (6)
O*W*2—H22⋯O3	0.89 (3)	1.95 (5)	2.745 (5)	149 (7)
O*W*3—H31⋯O7	0.90 (3)	1.97 (4)	2.834 (7)	162 (9)
O*W*3—H31⋯O8^iii^	0.90 (3)	2.53 (8)	3.141 (7)	126 (7)
O*W*3—H32⋯O9^iv^	0.89 (3)	2.49 (4)	3.360 (7)	166 (9)
O*W*3—H32⋯O9^v^	0.89 (3)	2.64 (9)	3.253 (8)	127 (8)

Symmetry codes: (i) 
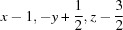
; (ii) 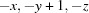; (iii) 
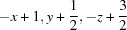
; (iv) 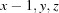; (v) 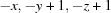.

**Table 3:** IR band assignments (cm^−1^) for YCu(TeO_3_)_2_(NO_3_)(H_2_O)_3_

Absorption bands	Assignment	
3460*w*	O—H stretch	
3145*w*	O—H stretch	
∼2900*w*	O—H stretch	
1755	H—O—H bend	
1645	H—O—H bend	
1605	H—O—H bend	
1345	ν_3_ anti­symmetric stretch NO_3_^−^	
1044	ν_1_ symmetric stretch NO_3_^−^	
734	ν_1_ (TeO_3_)^2−^ symmetric stretch	
636	ν_3_ (TeO_3_)^2−^ anti­symmetric stretch	
547	*M*—O lattice modes	
447	*M*—O lattice modes	

**Table 4:** Experimental details

Crystal data	
Chemical formula	YCu(TeO_3_)_2_(NO_3_)(H_2_O)_3_
*M*_r_	619.71
Crystal system, space group	Monoclinic, *P*2_1_/*c*
Temperature (K)	100
*a*, *b*, *c* (Å)	7.2560 (15), 20.654 (4), 7.0160 (14)
β (°)	94.63 (3)
*V* (Å^3^)	1048.0 (4)
*Z*	4
Radiation type	Synchrotron, λ = 0.71073 Å
μ (mm^−1^)	13.06
Crystal size (mm)	0.02 × 0.02 × 0.01

Data collection	
Diffractometer	ADSC Quantum 315r detector
Absorption correction	Multi-scan (*SADABS*; Bruker, 2001 ▸)
*T*_min_, *T*_max_	0.295, 0.433
No. of measured, independent and observed [*I* > 2σ(*I*)] reflections	20336, 2901, 2810
*R*_int_	0.054
(sin θ/λ)_max_ (Å^−1^)	0.704

Refinement	
*R*[*F* ^2^ > 2σ(*F* ^2^)], *wR*(*F* ^2^), *S*	0.033, 0.074, 1.14
No. of reflections	2901
No. of parameters	173
No. of restraints	6
H-atom treatment	Only H-atom coordinates refined
Δρ_max_, Δρ_min_ (e Å^−3^)	1.45, −1.56

Computer programs: local program, *XDS* (Kabsch, 2010 ▸), *XPREP* (Bruker, 2001 ▸), *SHELXS97* (Sheldrick, 2008 ▸), *SHELXL2014* (Sheldrick, 2015 ▸), *CrystalMaker* (Palmer, 2009 ▸) and *publCIF* (Westrip, 2010 ▸).

## supplementary crystallographic information

### Crystal data

**Table:**

YCu(TeO_3_)_2_(NO_3_)(H_2_O)_3_	*F*(000) = 1124
*M**_r_* = 619.71	*D*_x_ = 3.928 Mg m^−^^3^
Monoclinic, *P*2_1_/*c*	Synchrotron radiation, λ = 0.71073 Å
*a* = 7.2560 (15) Å	Cell parameters from 20243 reflections
*b* = 20.654 (4) Å	θ = 2.8–30.0°
*c* = 7.0160 (14) Å	µ = 13.06 mm^−^^1^
β = 94.63 (3)°	*T* = 100 K
*V* = 1048.0 (4) Å^3^	Prism, dark blue
*Z* = 4	0.02 × 0.02 × 0.01 mm

### Data collection

**Table:**

ADSC Quantum 315r detector diffractometer	2810 reflections with *I* > 2σ(*I*)
Radiation source: synchrotron	*R*_int_ = 0.054
φ scan	θ_max_ = 30.0°, θ_min_ = 2.8°
Absorption correction: multi-scan (*SADABS*; Bruker, 2001)	*h* = −10→10
*T*_min_ = 0.295, *T*_max_ = 0.433	*k* = −29→29
20336 measured reflections	*l* = −9→9
2901 independent reflections	360 standard reflections every 1 reflections

### Refinement

**Table:**

Refinement on *F*^2^	Hydrogen site location: difference Fourier map
Least-squares matrix: full	Only H-atom coordinates refined
*R*[*F*^2^ > 2σ(*F*^2^)] = 0.033	*w* = 1/[σ^2^(*F*_o_^2^) + 11.0043*P*] where *P* = (*F*_o_^2^ + 2*F*_c_^2^)/3
*wR*(*F*^2^) = 0.074	(Δ/σ)_max_ = 0.001
*S* = 1.14	Δρ_max_ = 1.45 e Å^−^^3^
2901 reflections	Δρ_min_ = −1.56 e Å^−^^3^
173 parameters	Extinction correction: SHELXL2014 (Sheldrick, 2015), Fc^*^=kFc[1+0.001xFc^2^λ^3^/sin(2θ)]^-1/4^
6 restraints	Extinction coefficient: 0.0094 (5)

### Special details

**Table:**

Geometry. All esds (except the esd in the dihedral angle between two l.s. planes) are estimated using the full covariance matrix. The cell esds are taken into account individually in the estimation of esds in distances, angles and torsion angles; correlations between esds in cell parameters are only used when they are defined by crystal symmetry. An approximate (isotropic) treatment of cell esds is used for estimating esds involving l.s. planes.

### Fractional atomic coordinates and isotropic or equivalent isotropic displacement parameters (Å^2^)

**Table:**

	*x*	*y*	*z*	*U*_iso_*/*U*_eq_	
Te1	0.21415 (4)	0.34749 (2)	0.49104 (4)	0.00849 (9)	
Te2	0.71124 (4)	0.15281 (2)	1.03341 (4)	0.00846 (9)	
Y1	−0.03880 (6)	0.28786 (2)	0.01247 (6)	0.00865 (11)	
Cu1	0.46115 (7)	0.24947 (2)	0.76001 (8)	0.00911 (12)	
N1	0.8271 (7)	0.0010 (2)	1.1795 (7)	0.0209 (9)	
O1	0.2531 (4)	0.26068 (15)	0.5797 (5)	0.0105 (6)	
O2	0.0133 (4)	0.31345 (15)	0.3301 (5)	0.0106 (6)	
O3	0.3929 (4)	0.34159 (15)	0.3116 (5)	0.0112 (6)	
O4	0.6704 (4)	0.23870 (15)	0.9406 (5)	0.0114 (6)	
O5	0.9077 (4)	0.18938 (15)	1.1933 (5)	0.0106 (6)	
O6	0.5292 (5)	0.15853 (15)	1.2113 (5)	0.0099 (6)	
O7	0.0622 (7)	0.45346 (18)	0.3091 (7)	0.0279 (10)	
O8	0.8860 (6)	0.05534 (18)	1.2373 (6)	0.0233 (8)	
O9	0.3327 (6)	0.49362 (19)	0.3909 (7)	0.0284 (9)	
OW1	−0.2697 (5)	0.36731 (17)	0.0483 (5)	0.0134 (6)	
H11	−0.266 (10)	0.4092 (16)	0.080 (10)	0.020*	
H12	−0.360 (8)	0.364 (4)	−0.046 (8)	0.020*	
OW2	0.1872 (5)	0.36858 (17)	−0.0246 (5)	0.0153 (7)	
H21	0.155 (10)	0.407 (2)	−0.070 (10)	0.023*	
H22	0.279 (8)	0.371 (4)	0.066 (8)	0.023*	
OW3	−0.2844 (7)	0.5007 (2)	0.1525 (8)	0.0317 (10)	
H31	−0.182 (9)	0.490 (4)	0.225 (12)	0.048*	
H32	−0.374 (10)	0.503 (4)	0.232 (12)	0.048*	

### Atomic displacement parameters (Å^2^)

**Table:**

	*U*^11^	*U*^22^	*U*^33^	*U*^12^	*U*^13^	*U*^23^
Te1	0.00827 (14)	0.00782 (13)	0.00912 (16)	−0.00013 (8)	−0.00084 (9)	−0.00064 (9)
Te2	0.00858 (14)	0.00783 (14)	0.00876 (16)	0.00007 (8)	−0.00061 (9)	−0.00066 (9)
Y1	0.00832 (19)	0.00855 (19)	0.0089 (2)	0.00012 (13)	−0.00055 (14)	0.00008 (13)
Cu1	0.0081 (2)	0.0088 (2)	0.0100 (3)	−0.00025 (17)	−0.00157 (18)	0.00098 (18)
N1	0.027 (2)	0.0128 (19)	0.022 (2)	−0.0008 (16)	−0.0056 (18)	0.0026 (16)
O1	0.0103 (14)	0.0089 (13)	0.0119 (16)	0.0001 (11)	−0.0018 (11)	0.0007 (11)
O2	0.0089 (14)	0.0102 (14)	0.0125 (16)	−0.0020 (11)	−0.0009 (11)	0.0003 (11)
O3	0.0080 (14)	0.0096 (13)	0.0163 (17)	0.0003 (11)	0.0032 (12)	0.0002 (11)
O4	0.0098 (14)	0.0093 (13)	0.0147 (17)	0.0005 (11)	−0.0018 (12)	0.0038 (11)
O5	0.0109 (14)	0.0095 (13)	0.0109 (16)	−0.0014 (11)	−0.0015 (11)	−0.0007 (11)
O6	0.0115 (14)	0.0116 (14)	0.0067 (15)	0.0012 (11)	0.0017 (11)	−0.0014 (11)
O7	0.038 (2)	0.0108 (16)	0.032 (2)	−0.0057 (16)	−0.0148 (19)	0.0042 (15)
O8	0.034 (2)	0.0136 (16)	0.020 (2)	0.0006 (15)	−0.0072 (16)	−0.0014 (14)
O9	0.026 (2)	0.0194 (18)	0.038 (3)	0.0027 (15)	−0.0085 (18)	0.0027 (17)
OW1	0.0137 (15)	0.0128 (15)	0.0128 (17)	0.0022 (12)	−0.0036 (12)	−0.0031 (12)
OW2	0.0176 (16)	0.0140 (15)	0.0137 (18)	−0.0029 (13)	−0.0016 (13)	0.0024 (12)
OW3	0.039 (3)	0.025 (2)	0.031 (3)	0.0023 (19)	0.000 (2)	0.0002 (18)

### Geometric parameters (Å, º)

**Table:**

Te1—O3	1.883 (3)	O2—Cu1^i^	3.571 (3)
Te1—O2	1.905 (3)	O3—Cu1^i^	1.986 (3)
Te1—O1	1.911 (3)	O3—Te2^i^	2.681 (3)
Te1—O6^i^	2.657 (3)	O4—Y1^ix^	2.359 (3)
Te1—O7	2.722 (4)	O4—Cu1^iii^	2.817 (4)
Te1—O5^ii^	2.837 (3)	O4—Te2^i^	3.661 (3)
Te2—O6	1.893 (3)	O4—Te1^iii^	3.801 (3)
Te2—O5	1.898 (3)	O4—Y1^iv^	3.847 (4)
Te2—O4	1.905 (3)	O5—Y1^x^	2.290 (3)
Te2—O3^iii^	2.681 (4)	O5—Y1^ix^	2.445 (3)
Te2—O8	2.723 (4)	O5—Te1^iv^	2.837 (3)
Te2—O2^iv^	2.798 (3)	O5—Cu1^iii^	3.543 (3)
Y1—O5^v^	2.290 (3)	O6—Cu1^iii^	1.999 (3)
Y1—O2	2.292 (3)	O6—Te1^iii^	2.657 (3)
Y1—O1^i^	2.357 (3)	O6—Y1^x^	3.801 (3)
Y1—O4^vi^	2.359 (3)	O7—N1^xi^	1.267 (6)
Y1—OW2	2.367 (4)	O7—Te2^ii^	3.798 (5)
Y1—OW1	2.373 (3)	O8—Te1^iv^	3.653 (5)
Y1—O5^vi^	2.445 (3)	O8—Y1^x^	3.787 (4)
Y1—O2^i^	2.497 (3)	O9—N1^xi^	1.233 (6)
Cu1—O1	1.904 (3)	O9—Te2^xi^	3.350 (4)
Cu1—O4	1.910 (3)	O9—Te2^i^	4.153 (4)
Cu1—O3^iii^	1.986 (3)	OW1—Te2^ii^	3.442 (4)
Cu1—O6^i^	1.999 (3)	OW1—Cu1^ii^	3.508 (4)
Cu1—O1^iii^	2.811 (4)	OW1—Te2^v^	3.628 (4)
Cu1—O4^i^	2.817 (4)	OW1—Cu1^vi^	3.632 (4)
N1—O9^vii^	1.233 (6)	OW1—H11	0.89 (3)
N1—O8	1.256 (6)	OW1—H12	0.89 (3)
N1—O7^vii^	1.267 (6)	OW2—Te1^xii^	3.447 (4)
N1—Te1^vii^	3.394 (4)	OW2—Cu1^xii^	3.574 (4)
O1—Y1^iii^	2.357 (3)	OW2—Cu1^i^	3.639 (4)
O1—Cu1^i^	2.811 (4)	OW2—H21	0.89 (3)
O1—Te1^iii^	3.679 (3)	OW2—H22	0.89 (3)
O1—Te2^i^	3.811 (3)	OW3—Te1^xiii^	4.018 (5)
O1—Y1^viii^	3.881 (4)	OW3—Te2^ii^	4.148 (5)
O2—Y1^iii^	2.497 (3)	OW3—H31	0.90 (3)
O2—Te2^ii^	2.798 (3)	OW3—H32	0.89 (3)
			
O3—Te1—O2	96.62 (15)	Cu1^i^—O3—Te2^i^	86.04 (11)
O3—Te1—O1	93.73 (14)	Te1—O3—Cu1	60.91 (10)
O2—Te1—O1	86.15 (14)	Cu1^i^—O3—Cu1	69.36 (10)
O3—Te1—O6^i^	77.24 (13)	Te2^i^—O3—Cu1	58.36 (7)
O2—Te1—O6^i^	155.35 (12)	Te1—O3—Y1	78.82 (10)
O1—Te1—O6^i^	70.67 (12)	Cu1^i^—O3—Y1	80.15 (10)
O3—Te1—O7	90.77 (15)	Te2^i^—O3—Y1	165.46 (11)
O2—Te1—O7	75.93 (13)	Cu1—O3—Y1	111.74 (8)
O1—Te1—O7	161.92 (13)	Te2—O4—Cu1	115.38 (17)
O6^i^—Te1—O7	127.41 (11)	Te2—O4—Y1^ix^	102.48 (14)
O3—Te1—O5^ii^	157.99 (12)	Cu1—O4—Y1^ix^	137.81 (17)
O2—Te1—O5^ii^	66.69 (13)	Te2—O4—Cu1^iii^	83.55 (12)
O1—Te1—O5^ii^	71.75 (12)	Cu1—O4—Cu1^iii^	93.83 (13)
O6^i^—Te1—O5^ii^	111.64 (10)	Y1^ix^—O4—Cu1^iii^	108.82 (13)
O7—Te1—O5^ii^	98.40 (13)	Te2—O4—Te2^i^	144.76 (16)
O6—Te2—O5	96.67 (15)	Cu1—O4—Te2^i^	61.40 (9)
O6—Te2—O4	94.00 (14)	Y1^ix^—O4—Te2^i^	77.07 (9)
O5—Te2—O4	85.42 (14)	Cu1^iii^—O4—Te2^i^	130.56 (10)
O6—Te2—O3^iii^	76.45 (13)	Te2—O4—Te1^iii^	69.18 (9)
O5—Te2—O3^iii^	153.70 (12)	Cu1—O4—Te1^iii^	57.63 (9)
O4—Te2—O3^iii^	70.03 (12)	Y1^ix^—O4—Te1^iii^	162.26 (14)
O6—Te2—O8	91.15 (14)	Cu1^iii^—O4—Te1^iii^	55.74 (6)
O5—Te2—O8	71.82 (13)	Te2^i^—O4—Te1^iii^	119.03 (9)
O4—Te2—O8	157.11 (13)	Te2—O4—Y1^iv^	93.11 (12)
O3^iii^—Te2—O8	132.81 (11)	Cu1—O4—Y1^iv^	87.45 (12)
O6—Te2—O2^iv^	159.67 (12)	Y1^ix^—O4—Y1^iv^	72.00 (9)
O5—Te2—O2^iv^	67.71 (13)	Cu1^iii^—O4—Y1^iv^	176.66 (11)
O4—Te2—O2^iv^	72.56 (12)	Te2^i^—O4—Y1^iv^	52.71 (5)
O3^iii^—Te2—O2^iv^	111.52 (10)	Te1^iii^—O4—Y1^iv^	122.87 (9)
O8—Te2—O2^iv^	95.81 (12)	Te2—O5—Y1^x^	136.03 (17)
O5^v^—Y1—O2	154.82 (12)	Te2—O5—Y1^ix^	99.64 (14)
O5^v^—Y1—O1^i^	111.21 (12)	Y1^x^—O5—Y1^ix^	108.37 (12)
O2—Y1—O1^i^	80.12 (12)	Te2—O5—Te1^iv^	100.31 (13)
O5^v^—Y1—O4^vi^	78.52 (12)	Y1^x^—O5—Te1^iv^	117.78 (13)
O2—Y1—O4^vi^	112.44 (12)	Y1^ix^—O5—Te1^iv^	78.46 (10)
O1^i^—Y1—O4^vi^	129.31 (11)	Te2—O5—Cu1^iii^	64.46 (9)
O5^v^—Y1—OW2	79.18 (12)	Y1^x^—O5—Cu1^iii^	83.26 (10)
O2—Y1—OW2	83.29 (12)	Y1^ix^—O5—Cu1^iii^	87.55 (9)
O1^i^—Y1—OW2	72.67 (12)	Te1^iv^—O5—Cu1^iii^	157.47 (12)
O4^vi^—Y1—OW2	153.52 (12)	Te2—O6—Cu1^iii^	111.53 (16)
O5^v^—Y1—OW1	84.04 (12)	Te2—O6—Te1^iii^	103.11 (14)
O2—Y1—OW1	78.45 (12)	Cu1^iii^—O6—Te1^iii^	86.06 (11)
O1^i^—Y1—OW1	154.67 (12)	Te2—O6—Cu1	61.11 (9)
O4^vi^—Y1—OW1	72.15 (12)	Cu1^iii^—O6—Cu1	69.15 (9)
OW2—Y1—OW1	91.49 (13)	Te1^iii^—O6—Cu1	58.27 (7)
O5^v^—Y1—O5^vi^	131.04 (10)	Te2—O6—Y1^x^	78.26 (10)
O2—Y1—O5^vi^	73.01 (11)	Cu1^iii^—O6—Y1^x^	80.34 (10)
O1^i^—Y1—O5^vi^	73.74 (11)	Te1^iii^—O6—Y1^x^	165.75 (11)
O4^vi^—Y1—O5^vi^	64.91 (11)	Cu1—O6—Y1^x^	112.14 (9)
OW2—Y1—O5^vi^	141.57 (12)	N1^xi^—O7—Te1	111.3 (3)
OW1—Y1—O5^vi^	112.16 (12)	N1^xi^—O7—Te2^ii^	149.8 (4)
O5^v^—Y1—O2^i^	72.04 (11)	Te1—O7—Te2^ii^	66.47 (9)
O2—Y1—O2^i^	132.19 (10)	N1^xi^—O7—Y1	140.9 (4)
O1^i^—Y1—O2^i^	64.87 (11)	Te1—O7—Y1	67.08 (8)
O4^vi^—Y1—O2^i^	72.51 (11)	Te2^ii^—O7—Y1	67.94 (6)
OW2—Y1—O2^i^	113.51 (12)	N1—O8—Te2	111.0 (3)
OW1—Y1—O2^i^	140.45 (11)	N1—O8—Te1^iv^	123.8 (4)
O5^vi^—Y1—O2^i^	66.79 (12)	Te2—O8—Te1^iv^	68.84 (9)
O1—Cu1—O4	179.67 (14)	N1—O8—Y1^x^	163.7 (4)
O1—Cu1—O3^iii^	92.32 (14)	Te2—O8—Y1^x^	71.18 (8)
O4—Cu1—O3^iii^	88.01 (14)	Te1^iv^—O8—Y1^x^	72.46 (7)
O1—Cu1—O6^i^	87.95 (13)	N1^xi^—O9—Te1	86.8 (3)
O4—Cu1—O6^i^	91.72 (14)	N1^xi^—O9—Te2^xi^	81.0 (3)
O3^iii^—Cu1—O6^i^	179.29 (14)	Te1—O9—Te2^xi^	148.43 (17)
O1—Cu1—O1^iii^	95.24 (13)	N1^xi^—O9—Te2^i^	140.3 (3)
O4—Cu1—O1^iii^	84.92 (13)	Te1—O9—Te2^i^	56.63 (6)
O3^iii^—Cu1—O1^iii^	68.03 (12)	Te2^xi^—O9—Te2^i^	138.25 (13)
O6^i^—Cu1—O1^iii^	111.29 (12)	Y1—OW1—Te2^ii^	96.11 (11)
O1—Cu1—O4^i^	84.84 (13)	Y1—OW1—Cu1^ii^	89.51 (11)
O4—Cu1—O4^i^	94.99 (13)	Te2^ii^—OW1—Cu1^ii^	55.27 (6)
O3^iii^—Cu1—O4^i^	112.68 (12)	Y1—OW1—Te2^v^	77.63 (10)
O6^i^—Cu1—O4^i^	68.00 (12)	Te2^ii^—OW1—Te2^v^	165.76 (11)
O1^iii^—Cu1—O4^i^	179.28 (9)	Cu1^ii^—OW1—Te2^v^	111.41 (9)
O9^vii^—N1—O8	121.6 (5)	Y1—OW1—Cu1^vi^	80.22 (9)
O9^vii^—N1—O7^vii^	120.0 (4)	Te2^ii^—OW1—Cu1^vi^	114.02 (10)
O8—N1—O7^vii^	118.3 (5)	Cu1^ii^—OW1—Cu1^vi^	58.82 (6)
O9^vii^—N1—Te2	77.9 (3)	Te2^v^—OW1—Cu1^vi^	52.63 (5)
O8—N1—Te2	48.7 (2)	Y1—OW1—H11	134 (5)
O7^vii^—N1—Te2	151.4 (4)	Te2^ii^—OW1—H11	83 (5)
O9^vii^—N1—Te1^vii^	71.9 (3)	Cu1^ii^—OW1—H11	125 (5)
O8—N1—Te1^vii^	165.1 (4)	Te2^v^—OW1—H11	111 (5)
O7^vii^—N1—Te1^vii^	48.4 (2)	Cu1^vi^—OW1—H11	142 (5)
Te2—N1—Te1^vii^	138.27 (15)	Y1—OW1—H12	110 (5)
Cu1—O1—Te1	114.77 (16)	Te2^ii^—OW1—H12	129 (5)
Cu1—O1—Y1^iii^	137.13 (17)	Cu1^ii^—OW1—H12	82 (5)
Te1—O1—Y1^iii^	103.12 (14)	Te2^v^—OW1—H12	45 (5)
Cu1—O1—Cu1^i^	94.20 (13)	Cu1^vi^—OW1—H12	37 (5)
Te1—O1—Cu1^i^	83.41 (12)	H11—OW1—H12	106 (6)
Y1^iii^—O1—Cu1^i^	109.90 (13)	Y1—OW2—Te1^xii^	96.56 (11)
Cu1—O1—Te1^iii^	60.78 (9)	Y1—OW2—Cu1^xii^	88.60 (11)
Te1—O1—Te1^iii^	143.74 (15)	Te1^xii^—OW2—Cu1^xii^	54.44 (6)
Y1^iii^—O1—Te1^iii^	76.93 (9)	Y1—OW2—Te1	77.77 (10)
Cu1^i^—O1—Te1^iii^	131.38 (10)	Te1^xii^—OW2—Te1	164.50 (12)
Cu1—O1—Te2^i^	57.54 (9)	Cu1^xii^—OW2—Te1	110.57 (10)
Te1—O1—Te2^i^	68.88 (9)	Y1—OW2—Cu1^i^	79.66 (10)
Y1^iii^—O1—Te2^i^	163.46 (14)	Te1^xii^—OW2—Cu1^i^	112.61 (10)
Cu1^i^—O1—Te2^i^	55.85 (6)	Cu1^xii^—OW2—Cu1^i^	58.20 (6)
Te1^iii^—O1—Te2^i^	118.32 (9)	Te1—OW2—Cu1^i^	52.42 (5)
Cu1—O1—Y1^viii^	87.20 (12)	Y1—OW2—H21	121 (5)
Te1—O1—Y1^viii^	92.47 (11)	Te1^xii^—OW2—H21	78 (5)
Y1^iii^—O1—Y1^viii^	71.31 (9)	Cu1^xii^—OW2—H21	128 (5)
Cu1^i^—O1—Y1^viii^	175.87 (11)	Te1—OW2—H21	117 (5)
Te1^iii^—O1—Y1^viii^	52.60 (5)	Cu1^i^—OW2—H21	157 (5)
Te2^i^—O1—Y1^viii^	122.26 (9)	Y1—OW2—H22	117 (5)
Te1—O2—Y1	136.04 (17)	Te1^xii^—OW2—H22	128 (5)
Te1—O2—Y1^iii^	98.37 (14)	Cu1^xii^—OW2—H22	86 (5)
Y1—O2—Y1^iii^	106.57 (12)	Te1—OW2—H22	47 (5)
Te1—O2—Te2^ii^	101.48 (14)	Cu1^i^—OW2—H22	46 (5)
Y1—O2—Te2^ii^	118.61 (13)	H21—OW2—H22	112 (7)
Y1^iii^—O2—Te2^ii^	77.90 (9)	Te1^xiii^—OW3—Te2^ii^	101.69 (12)
Te1—O2—Cu1^i^	63.55 (9)	Te1^xiii^—OW3—H31	77 (6)
Y1—O2—Cu1^i^	82.11 (10)	Te2^ii^—OW3—H31	59 (6)
Y1^iii^—O2—Cu1^i^	86.71 (9)	Te1^xiii^—OW3—H32	69 (6)
Te2^ii^—O2—Cu1^i^	156.91 (12)	Te2^ii^—OW3—H32	66 (6)
Te1—O3—Cu1^i^	112.24 (16)	H31—OW3—H32	106 (9)
Te1—O3—Te2^i^	102.52 (15)		

Symmetry codes: (i) *x*, −*y*+1/2, *z*−1/2; (ii) *x*−1, −*y*+1/2, *z*−1/2; (iii) *x*, −*y*+1/2, *z*+1/2; (iv) *x*+1, −*y*+1/2, *z*+1/2; (v) *x*−1, −*y*+1/2, *z*−3/2; (vi) *x*−1, *y*, *z*−1; (vii) −*x*+1, *y*−1/2, −*z*+3/2; (viii) *x*, *y*, *z*+1; (ix) *x*+1, *y*, *z*+1; (x) *x*+1, −*y*+1/2, *z*+3/2; (xi) −*x*+1, *y*+1/2, −*z*+3/2; (xii) *x*, *y*, *z*−1; (xiii) −*x*, −*y*+1, −*z*+1.

### Hydrogen-bond geometry (Å, º)

**Table:**

*D*—H···*A*	*D*—H	H···*A*	*D*···*A*	*D*—H···*A*
O*W*1—H11···O*W*3	0.89 (3)	1.96 (3)	2.854 (6)	174 (7)
O*W*1—H12···O6^v^	0.89 (3)	1.88 (4)	2.729 (5)	157 (7)
O*W*2—H21···O8^v^	0.89 (3)	2.41 (6)	3.074 (5)	132 (6)
O*W*2—H21···O*W*3^xiv^	0.89 (3)	2.22 (5)	2.949 (6)	139 (6)
O*W*2—H22···O3	0.89 (3)	1.95 (5)	2.745 (5)	149 (7)
O*W*3—H31···O7	0.90 (3)	1.97 (4)	2.834 (7)	162 (9)
O*W*3—H31···O8^xi^	0.90 (3)	2.53 (8)	3.141 (7)	126 (7)
O*W*3—H32···O9^xv^	0.89 (3)	2.49 (4)	3.360 (7)	166 (9)
O*W*3—H32···O9^xiii^	0.89 (3)	2.64 (9)	3.253 (8)	127 (8)

Symmetry codes: (v) *x*−1, −*y*+1/2, *z*−3/2; (xi) −*x*+1, *y*+1/2, −*z*+3/2; (xiii) −*x*, −*y*+1, −*z*+1; (xiv) −*x*, −*y*+1, −*z*; (xv) *x*−1, *y*, *z*.
