# Supplementary figures and images for: YCu(TeO3)2(NO3)(H2O)3: a novel layered tellurite
Source: Acta Crystallogr E Crystallogr Commun. 2016 Jul 19;72(Pt 8):1138–42. doi: 10.1107/S2056989016011464 (PMC4971857; doi:10.1107/S2056989016011464)

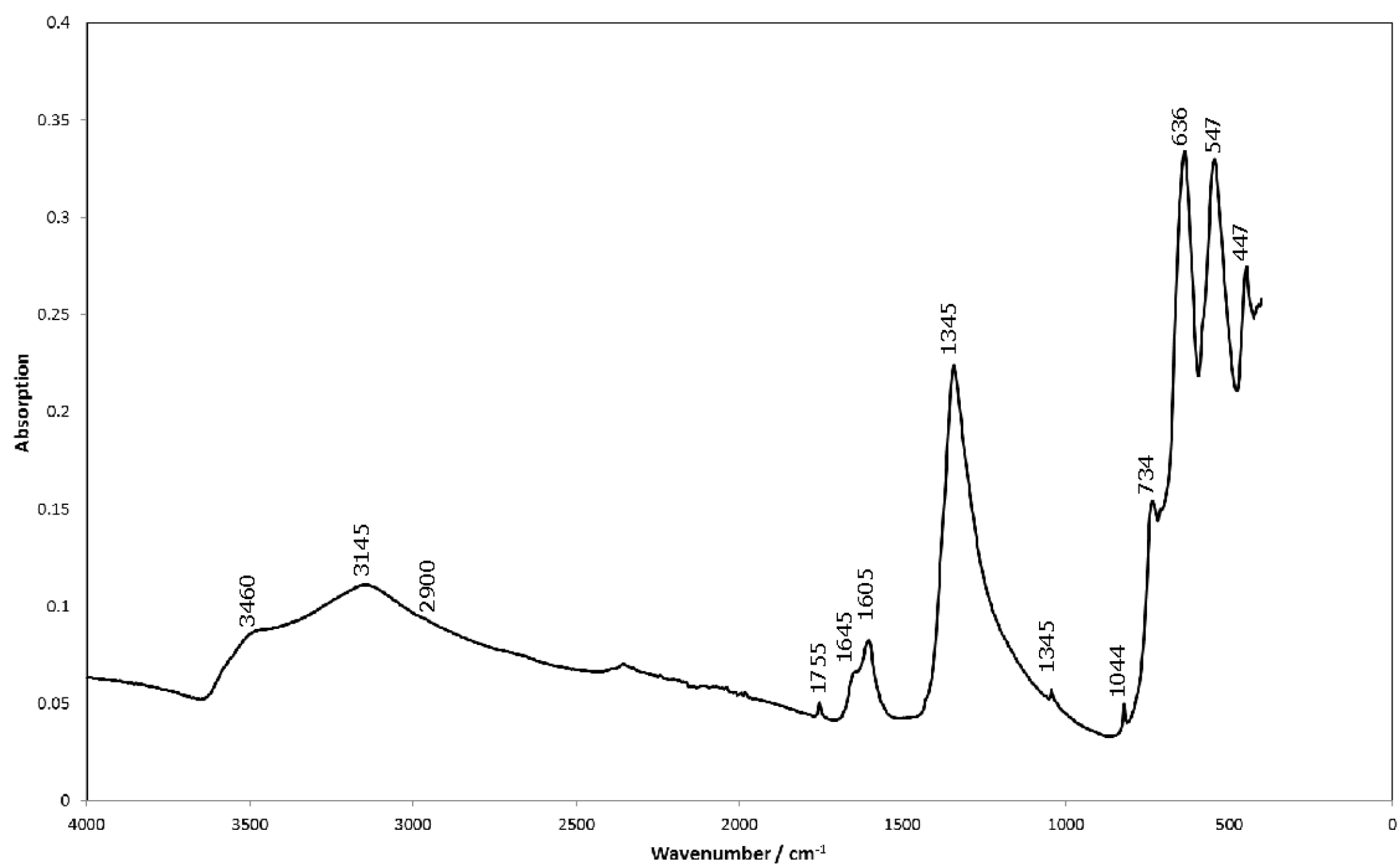

Supplement: Supplementary file 3 [file e-72-01138-sup3.pdf]
